# IR sensitivity enhancement of CMOS Image Sensor with diffractive light trapping pixels

**DOI:** 10.1038/s41598-017-04200-y

**Published:** 2017-06-19

**Authors:** Sozo Yokogawa, Itaru Oshiyama, Harumi Ikeda, Yoshiki Ebiko, Tomoyuki Hirano, Suguru Saito, Takashi Oinoue, Yoshiya Hagimoto, Hayato Iwamoto

**Affiliations:** Sony Semiconductor Solutions Corporation, Atsugi Tec., 4-14-1 Asahi-cho, Atsugi, Kanagawa 243-0014 Japan

## Abstract

We report on the IR sensitivity enhancement of back-illuminated CMOS Image Sensor (BI-CIS) with 2-dimensional diffractive inverted pyramid array structure (IPA) on crystalline silicon (c-Si) and deep trench isolation (DTI). FDTD simulations of semi-infinite thick c-Si having 2D IPAs on its surface whose pitches over 400 nm shows more than 30% improvement of light absorption at λ = 850 nm and the maximum enhancement of 43% with the 540 nm pitch at the wavelength is confirmed. A prototype BI-CIS sample with pixel size of 1.2 μm square containing 400 nm pitch IPAs shows 80% sensitivity enhancement at λ = 850 nm compared to the reference sample with flat surface. This is due to diffraction with the IPA and total reflection at the pixel boundary. The NIR images taken by the demo camera equip with a C-mount lens show 75% sensitivity enhancement in the λ = 700–1200 nm wavelength range with negligible spatial resolution degradation. Light trapping CIS pixel technology promises to improve NIR sensitivity and appears to be applicable to many different image sensor applications including security camera, personal authentication, and range finding Time-of-Flight camera with IR illuminations.

## Introduction

In the past decade, c-Si based CISs have rapidly replaced CCD image sensors in most commercial camera applications, due to their superiority in power consumption and higher flexibility readout. Now, most smart-phones and personal mobile computers use one or more camera modules with CIS, and CIS dominates the image sensor market. CIS technology has drastically improved its image quality along with shrinking pixel size, increasing sensor resolution, and adding smart analog readout circuit and sophisticated signal processing. The evolution of CIS architecture has also included improvements such as back-illuminated (BI) technology and imager + logic stack technologies^1–3^. The trend of shrinking pixel size continue and now a commercially available pixels have been shrunk to 1.0 μm square^4^. To maintain high sensitivity with such small pixels, BI-CIS technology has been widely utilized, because it is free from design restriction of tightly packed metal wires for signal readout. This make it possible to maintain a high fill-factor compared to the front side illuminated CISs (FI-CISs). De-facto standard color CISs use RGB on-chip color filter on each pixel in Bayer pattern, which consists of a 2 × 2 color unit cell with two green filters in the diagonal positions and blue and red in the off-diagonal positions^5^. BI-CISs have a photo-absorption layer with typical Si thickness of 3 μm. This thickness is optimized to absorb visible light with acceptable lateral color-crosstalk among adjacent pixels^6^. On the other hand, demand of non-visible, especially near-infrared (NIR) sensitive image sensors for security, personal authentication, and range finding applications has been growing. To make cutting-edge BI-CISs applicable for NIR use, it is important to enhance NIR sensitivity. The most straightforward approach to improve NIR sensitivity is simply to make the photo absorption layer thicker. But this degrades the visible image quality due to the lateral crosstalk especially for small pixel sizes. In addition, chip cost is increased because fabrication of thick substrate BI-CISs requires huge capital investments in manufacturing equipment typified by high energy ion implanters.

It is well known that black silicon, which has random needle-shaped surface, has very low reflectivity and high absorption efficiency^7^. These structures have been applied to c-Si solar cells and image sensors to improve photo-electro conversion efficiencies and sensor sensitivities^8, 9^. However, the needle-shaped surface has issues when applied to consumer image sensors, which have small pixel sizes. The random surface structure is typically a few 100 nm in size. This means that each pixel contains random but limited number of structures, and hence, it results in large deviation of pixel characteristics such as PRNU (photo response non-uniformity), which is not good for high quality, uniform 2D imaging capabilities.

Another surface structure has been reported with carefully designed uneven and periodic surface for thin-film c-Si solar cell. The c-Si solar cell was designed with the (100) surface containing wavelength size-scale inverted pyramid array (IPA) structures, composed of (111) facet on (100). It was reported that the amount of absorption was enhanced and its figure of merit (FOM) approached to 4*n* ^2^ ^10–12^. If the typical size of the uneven structure on c-Si surface is much smaller than the wavelength of interest, the effective refractive index between c-Si and the upper medium has an intermediate value, which is well-known as a moth-eye anti-reflection coating or refractive index gradient structure^13^. Meanwhile, in case that the size is comparable to the wavelength of interest, it is expected to have strong diffraction as well as anti-reflection effect within the c-Si, extending the optical path length within c-Si. This will result in considerable absorption enhancement, which is directly proportional to sensor sensitivity and quantum efficiency (QE). In this paper, we propose the novel pixel architecture of BI-CISs with IPA structure on c-Si surface plus deep trench isolations (DTI) between pixels to enhance NIR sensor sensitivity.

## Results

### Design concept of BI-CIS pixels

**Figure 1:**
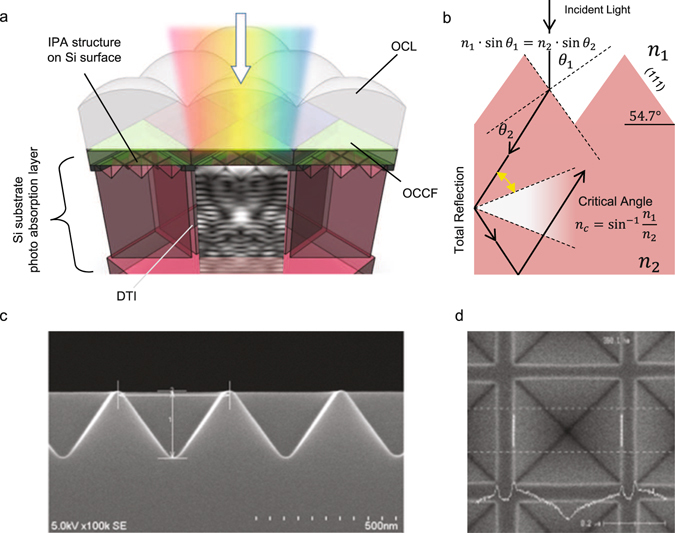
(**a**) A schematic image of the prototype BI-CIS pixels with the inverted pyramid array structures (IPA) and deep trench isolation (DTI). Each pixel equips on-chip micro lens (OCL) and RGB on-chip color filter (OCCF) in Bayer format. At the bottom of each pixel, there are CMOS transistors to readout collected carriers, which are not illustrated for simplicity. (**b**) A schematics of light rapping pixel concept. Normal incident light has the incident angle *θ*_1_. It is refracted with the angle of *θ*_2_ following Snell’s law. The refracted light is totally reflected at the pixel sidewall in case that the angle is larger than the critical angle. As a result, the effective optical path length elongate with in the Si substrate. (**c**,**d**) SEM cross-section and top-view images of c-Si with the IPA.

We report on the NIR sensitivity enhancement of BI-CIS with the IPA structures on c-Si surface. Additionally, lateral crosstalk is suppressed with the use of low refractive index DTI between pixels. CIS has a densely packed 2D pixel array with pixel size less than few μm square to enable high spatial resolution images with small camera module size for cost-competitive products. However, an uneven surface with IPAs will diffract the incident light and degrade the spatial resolution due to the strong diffraction within the substrate. To compensate this feature but maintain the effectiveness of absorption enhancement, we employed DTI between pixels. DTI minimizes lateral photon crosstalk by confining the incident light within the pixel. This is accomplished by the use of low refractive index DTI fill, which enables total reflection between c-Si photo-detector and SiO_2_. We illustrate the schematic of BI-CIS pixels which have the IPA surface and DTI in Fig. 1a. Each pixel has an on-chip micro lens (OCL) to maximize fill-factor and sensor sensitivity as well as on-chip color filters (OCCFs) to selectively collect RGB color intensity information. A brief working principle for sensitivity enhancement is described in the Methods section (1).

### Simulation for IPA size optimization

**Figure 2:**
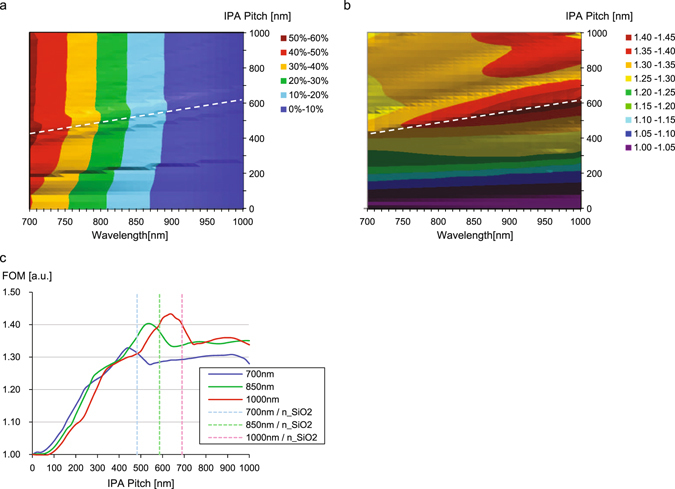
(**a**) Amount of light absorption of semi-infinite c-Si substrate with IPA by FDTD simulation. The horizontal axis is the wavelength of the incident light. The vertical axis is the pitch of IPA. Color indicates amount of the absorption of the c-Si which integrated from the surface to 3 μm depth. (**b**) Normalized amount of light absorption (Figure of Merit) which is divided by the amount of absorption with flat surface case (corresponding to 0 nm pitch). White dashed lines in panels (**a**) and (**b**), the local maximum at each wavelength, slightly depends on the wavelength. (**c**) Vertical cuts of (**b**) at the wavelengths of 700 nm, 850 nm and 1000 nm. Light colored dashed lines are the effective wavelengths in SiO_2_. Here, the refractive index is assumed to 1.45.

At first, we performed the FDTD simulations of bulk c-Si to evaluate effectiveness of the structure. Here, c-Si is assumed to have semi-infinite thickness in the Z-direction, so that there is no reflection or diffraction from the bottom boundary of c-Si, and hence, it is possible to extract pure contribution of the diffractive surface only. We assumed SiO_2_ buffer layer on the c-Si layer. Figure 2a shows the simulation results. The horizontal axis is the wavelength of incident light and the vertical axis is the pitch of the IPA structure. The color corresponds to the amount of absorption at each wavelength. The graph shows absorption enhancement as the pitch increases. The enhancement has plateaus over a pitch size of around 500 nm. The simulated absorption of 3 μm thick c-Si at 850 nm is calculated to be 12.5% with flat surface versus up to 16.5% with the IPA surface pitch of 400 nm (a 31% improvement). Figure 2b is the normalized absorption enhancement, FOM, defined as IPA surface absorption divided by the flat surface absorption at each wavelength. It is clearly seen that fine features exist in the graph. The most prominent one is that the white-dashed line has a local maximum at each wavelength. It has slight wavelength dependency and suggests that a 400 nm pitch is good for enhancement at λ = 700 nm sensitivity while the 600 nm pitch is good for λ = 950 nm. At λ = 850 nm, the best pitch is estimated to 540 nm and it has 18.1% absorption, corresponding to a FOM of 43%. Figure 2c shows the vertical cuts of Fig. 2b at λ = 700 nm, 850 nm and 1000 nm, respectively. Here, these wavelengths correspond to the effective wavelengths in the medium, λ_n_SiO2_ = 483 nm, 586 nm and 690 nm, respectively. They are slightly larger than the optimal pitch of 440 nm, 540 nm and 640 nm at each wavelength, suggesting that the optimal IPA pitch and local absorption enhancement is related to Wood’s anomaly. It is also worthwhile to note that the aspect ratio of the IPA structure is fixed to 0.708 (=1/tan (54.7°) because of the facet angle between c-Si (111) and (100).

**Figure 3:**
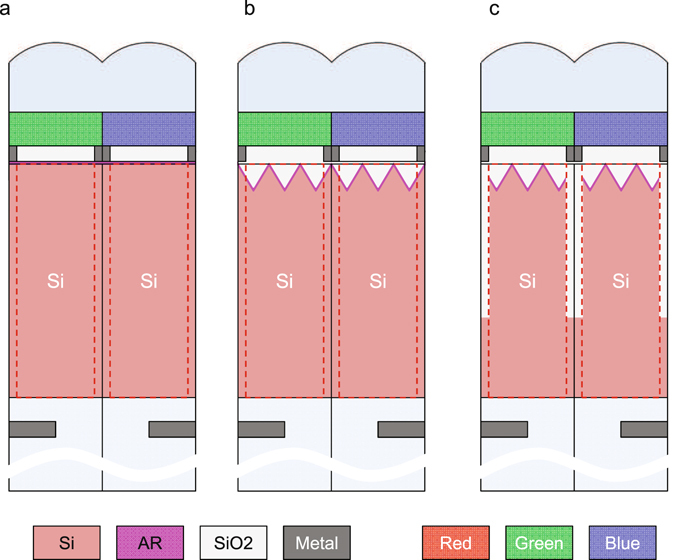
(**a**) A schematics of the reference sample which has flat Si surface with AR coating (Sample A). The thickness of Si photo detector is 3 μm. The on-chip micro lens height is 1.7 μm from the Si surface to the top with the curvature radius of 0.85 μm. Each pixel equips on-chip color filter whose thickness of 0.5 μm. (**b**) A schematics of Sample B, which is same as Sample A, but with the IPA structure with the pitch of 400 nm, so that each pixel contains 3 × 3 IPA. (**c**) A schematics of Sample C which is same as Sample B except the DTI whose width of 200 nm and depth of 2000 nm.

**Figure 4:**
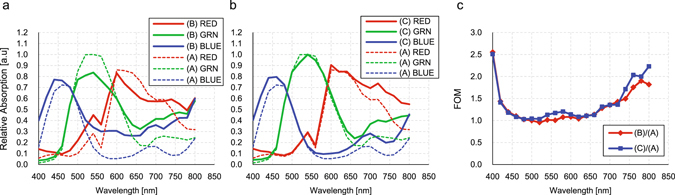
(**a**) FDTD simulation results of Samples A and B shown in Fig. 3. Dashed RGB lines show the normalized amount of the light absorptions of RGB pixels of Sample A. Here the amount of peak absorption of green pixel is assumed to be 1.0 for normalization. Solid RGB lines show the amount of light absorption of RGB pixels of sample B. (**b**) Simulation results of Sample A with dashed lines and sample C with solid lines. (**c**) Figure of Merit, the normalized amount of absorption by sample A. The red diamond is the ratio between sample B and sample A, and the blue square is the ratio between sample C and sample A.

Next, we performed simulation with the BI-CIS pixel model and its schematics are shown in Fig. 3. We assumed a 1.2 μm square pixel with 3 μm thick c-Si photo-detector and on-chip color filter and micro lens. The simulation conditions are written in Methods section (2). Here, Fig. 3a is a schematic of a typical BI-CIS pixel for the reference (hereinafter Sample A), and Fig. 3b is with 400 nm pitch IPA on c-Si surface (Sample B). Figure 3c is the same as B, but with 2 μm deep DTI (Sample C), so that each pixel of Sample B and C contains 3 × 3 IPA. The simulated results are plotted in Fig. 4. In each plot, the RGB color lines correspond to the pixel sensitivity with RGB on-chip color filters. The simulation results of Sample A are plotted in dashed lines on the both figures. Here the peak Si absorption of green pixel of Sample A is set to be 1.0 for normalization. Figure 4a is the comparison of Samples A and B, showing Sample B has lower peak absorption of 0.80 as well as big spectral crosstalk due to the diffractive IPA surface. Figure 4b is that of Samples A and C, showing comparable peak absorption, but with spectral crosstalk suppressed with larger absorption enhancement from λ = 700 nm to 800 nm. Figure 4c is the FOM which is the ratio of the RGB integrated sensitivities of Samples B or C structure divided by the Sample A. This FOM shows a large sensitivity enhancement over 700 nm. The averaged FOM from λ = 700 nm to 800 nm is 1.63 for Sample B and 1.78 for Sample C, respectively. It is also important to point out that amount of absorption of blue pixel around λ = 400–450 nm is improved, suggesting the IPA structure is good not only for NIR sensitivity enhancement with light trapping but also good broadband anti-reflection coating.

### Device prototyping and the results

Following the simulation, we fabricated a 1/2.3 inch BI-CIS prototype which has 20 Mega pixels. A pixel size of the prototype BI-CIS is 1.20 μm square. The size/pitch of IPA is set to 400 nm so that each pixel contains 3 × 3 IPA. The sensor has 3 μm thick c-Si photo-detectors as well as on-chip micro lenses and RGB color filters for each 2 × 2 pixel unit in Bayer pattern as illustrated in Fig. 1a. For comparison, we prepared 3 types of samples as shown in Fig. 3, corresponding to the simulated structures of Sample A, Sample B and Sample C structures, respectively. Brief fabrication recipe of IPAs on BI-CIS are described in the Methods section (3).

**Figure 5:**
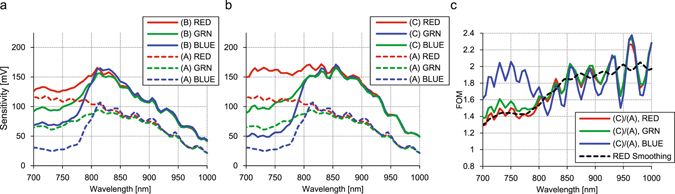
(**a**) Measured spectral sensitivities of the prototype samples A and B. Dashed lines are the sensor sensitivity of sample A for reference. Solid lines are those of sample B with 400 nm pitch IPA structure. (**b**) Dashed lines are data of sample A, and solid lines are those of sample C with the IPA structure and DTI. (**c**) Normalized sensor sensitivities of sample C divided with sample A sensitivities. RGB colors correspond to the RGB pixels. The black dashed line is the running average over 50 nm of the red pixel to make quite the ripple due to Fabry-Perot interference of bulk-Si.

Figure 5 shows the spectral sensitivity characteristics of 3 bare samples over the wavelength from 700 nm to 1000 nm in steps of 10 nm taken with collimated monochromatic illumination. The measured conditions are described in the Method section (4). Figure 5a shows the comparison between Sample A and Sample B, plotted as dashed lines and solid lines respectively. Figure 5b shows the comparison between Sample A in dashed lines and Sample C in the solid lines. Here RGB colors correspond to RGB pixels in both figures. Figure 5c plots the FOMs of RGB pixels which are the ratio of the sensor sensitivity between Sample C and Sample A. Although there are strong ripples in each color due to Fabry-Perot interference of the bulk Si, it is clearly seen that the FOM becomes larger as the wavelength increases, and the FOM is about 1.6 at 800 nm, 1.8 at 850 nm and 1.9 at 900 nm, respectively. The trend is clearly seen especially for red pixels, because the filter is almost transparent over 700 nm, while blue and green filters gradually become transparent over 700 nm. This difference mainly comes from the difference in absorption properties of the RGB filter dyes as well as the thickness of the on-chip color filters.

**Figure 6:**
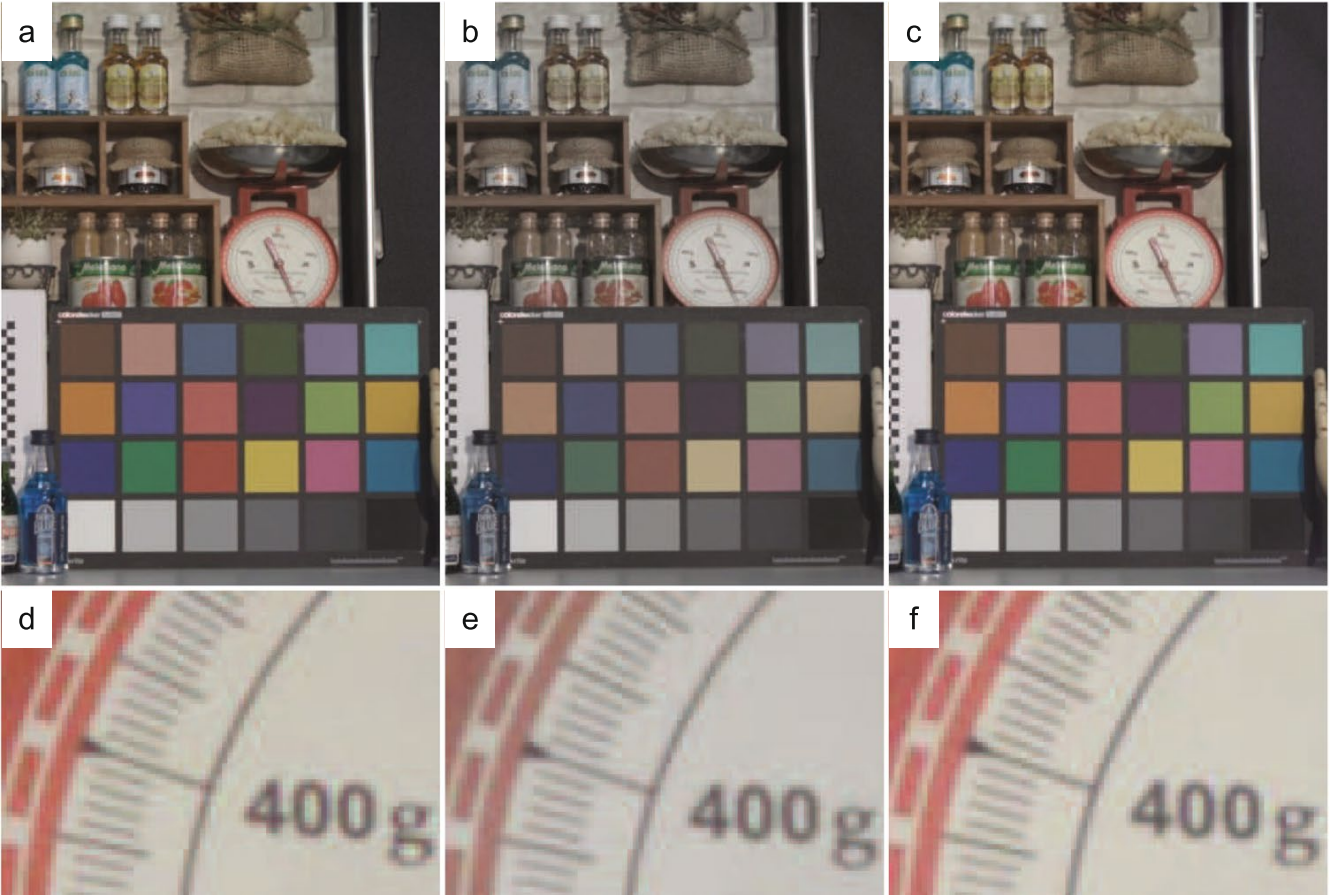
(**a**–**c**) Shows visible photographic images taken with the demo camera. An indoor scene including miscellaneous objects and the Macbeth 24 color chart (X-Rite, ColorChecker Classic) are taken with the camera with Tamron C-mount lens (focal length of 12.5 mm, F#2.8). (**a**) An image is taken with sample A. (**b**) An image is taken with sample B. (**c**) An image is taken with sample C. (**d**–**f**) Close up images of the red scale shown in center of the upper panels (**a**–**c**).

In Fig. 6, we show visible photographic images taken by the prototype samples with a Tamron C-mount lens (focal length 12.5 mm, F# 2.8). The image capturing conditions are as follows. Analog and digital gains are set to 0 dB. Exposure time is set to be 33 ms (corresponds to 30 fps), and illuminance level is about 1000 lux with a halogen lamp. Because c-Si based CISs have NIR sensitivity up to λ = 1100 nm, an IR cut filter (λ_cutoff_ = 750 nm) was put in front of the device. Then white-balancing was applied to make RGB levels equal using white-gray patches of the X-Rite ColorChecker chart. Additionally, gamma-correction was applied to make the linear sensor output more logarithmic like the human eye^14^. There is noticeable color change in Fig. 6b images taken by Sample B due to the considerable color crosstalk with the IPA structure, whereas the images in Fig. 6a taken by Sample A and Fig. 6c taken by Sample C are very similar with each other. This shows that spatial color crosstalk is mostly eliminated with the DTI. Figure 6d–f are the close up views of the red scale. The raw digit counts of the chart taken with each samples are summarized in Table 1. These counts are after application white-balancing but before gamma-correction, so that they are linearly proportional to light intensity. The data are averaged over 200 × 200 pixels on RGB and Gray patches on the chart. It is clearly shown that the RGB color pixel intensity ratio for Sample B has less contrast than those of Samples A and C, and gray patch signal levels of Sample C is larger than that of Samples A and B.

**Table 1:** RGB color pixel outputs of X-Rite, ColorChecker chart panels, as shown in Fig. 6.

Color	Sample A				Sample B				Sample C			
	Blue	Green	Red	Gary	Blue	Green	Red	Gary	Blue	Green	Red	Gary
Color ID	13	14	15	22	13	14	15	22	13	14	15	22
R pixel	136 ± 9	322 ± 12	845 ± 20	483 ± 15	150 ± 9	393 ± 13	670 ± 17	481 ± 15	144 ± 9	344 ± 13	909 ± 21	525 ± 16
G pixel	189 ± 10	560 ± 16	330 ± 13	483 ± 16	188 ± 10	507 ± 16	404 ± 14	482 ± 16	200 ± 10	585 ± 17	381 ± 14	524 ± 16
B pixel	411 ± 19	397 ± 18	257 ± 14	497 ± 20	302 ± 13	428 ± 15	354 ± 13	487 ± 16	427 ± 18	414 ± 17	317 ± 15	542 ± 19

The data unit is digital count after optical black level subtraction and white-balancing. The data are averaged over 200 × 200 pixels for each color panel.

**Figure 7:**
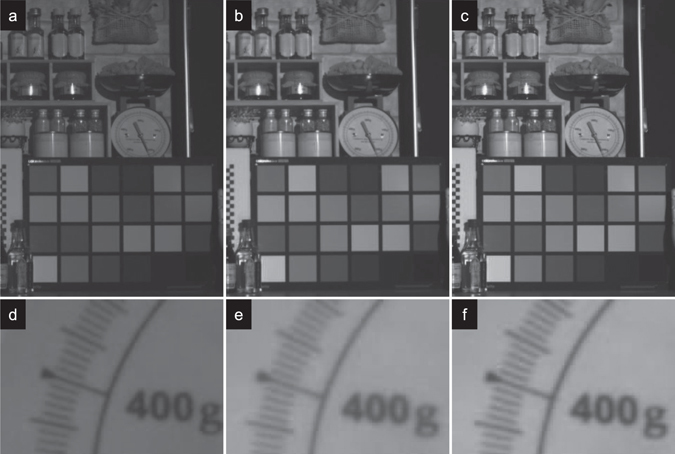
(**a**–**c**) Shows near-infrared images taken with the demo camera. An indoor scene is same to the visible images shown in Fig. 6. An IR pass filter which transmits only 700nm-1200nm is set in front of the lens. (**a**) A NIR image is taken with sample A. (**b**) A NIR is taken with sample B. (**c**) A NIR is taken with sample C. (**d**–**f**) Close up images of the scale shown in center of the upper panels (**a**–**c**).

In Fig. 7, we show NIR images of prototype samples with same optical setting except an IR pass filter is used instead of an IR-cut filter. Analog and digital gains are same as 0 dB, while exposure time is 33 mS. An IR band-pass filter that transmits only 700–1200 nm wavelength was put in front of the devices. The average signal counts of the white patch is 1712 ± 43 [digit], 2786 ± 54 [digit], 3001 ± 59 [digit] respectively. This results is a 75% sensor sensitivity enhancement achieved for Sample C compared to Sample A. Figure 7d–f are a close up views of the scale and the number “400” is sharpest for Sample A (d) whereas Sample C (f) is slightly worse than that of Sample A. It is also clearly seen that Sample B (e) has distinct degradation of spatial resolution with blur edges.

## Discussion

**Figure 8:**
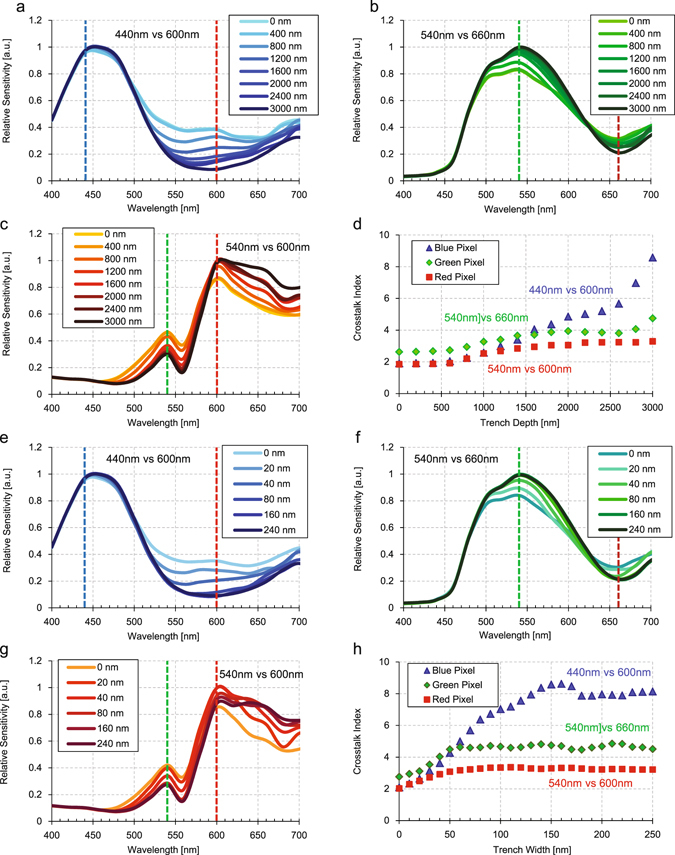
(**a**–**c**) These panels show the simulated spectral sensitivities of RGB pixels of sample C changing DTI depth from 0 nm (without DTI) to 3000 nm (DTI penetrate the substrate) in steps of 400 nm. (**a**) Shows the spectral sensitivity of blue pixel, (**b**) is that of green pixel, and (**c**) is that of red pixel. (**d**) This panel shows the spectral color ratio as index of photonic color crosstalk to adjacent pixels. Blue triangles are the ratio between 440 nm and 600 nm in blue pixel from (**a**), green diamonds are the ratio between 540 nm and 660 nm in green pixel from (**b**), and red squares are the ratio between 600 nm and 540 nm in red pixel from (**c**). (**e**–**g**) Show the same simulation results but changing DTI width from 0 nm to 240 nm with fixed depth of 3000 nm. (**e**) is for the blue pixel, (**f**) is for the green pixel, and (**g**) is for the red pixel. (**h**) This panel shows the spectral color ratio derived from (**e**–**g**) plotted in same fashion as panel (**d**).

The prototype measurements show there is considerable NIR sensitivity enhancement for the samples which have IPA and DTI, consistent with the FDTD simulations. In practical use, it is important to confirm how deep and thick a DTI is required to minimize the lateral crosstalk and spatial resolution degradation especially in RGB color imaging. Therefore FDTD simulation was performed by changing the DTI size parameter with Sample C structure. Figure 8a–d show the dependency of color crosstalk on DTI depth for each RGB pixel. Because the c-Si photodetector thickness is set to 3 μm, we performed the simulation by sweeping DTI depth from 0 nm to 3000 nm from the top-side of the pixel surface. It is assumed that the DTI width of 200 nm is enough thick to prevent near-field tunneling effect. Figure 8d is the summary of simulation. Here we introduce the color crosstalk indices of the intensity ratio of 440 nm vs 600 nm for blue pixels, that of 540 nm vs 660 nm for green pixels and that of 540 nm vs 600 nm for red pixels. Although the red pixel index (540/600) is saturated over 2200 nm, the green pixel index (540/660) is a maximum at 3000 nm depth and the blue index (440 nm/600 nm) rapidly increases over 2500 nm.These features suggest that a fully penetrated DTI is necessary to minimize the lateral crosstalk. Even a small space around the bottom boundary increases color crosstalk. Furthermore, the transmitted portion from backside of c-Si photo-detector might be diffracted by the metal wires or contact poly-Si gate structures and increase the color crosstalk.

Figure 8e–h show the dependency of color crosstalk on DTI width for each RGB pixels to confirm how wide DTI is required to trap the light within the pixel with the total reflection. Here we assume the DTI depth is the same as the c-Si photodetector for simplicity. We performed the simulation by sweeping DTI width from 0 nm to 250 nm. Figure 8h is the summary of the simulation. Here we use same crosstalk indices, the intensity ratio of 440 nm vs 600 nm for blue pixel, 540 nm vs 660 nm for green pixel and 540 nm vs 600 nm for red pixel. All RGB index are saturated over 150 nm suggesting that a DTI width over 150 nm is needed to minimize lateral color crosstalk within the c-Si. In our prototype samples, the DTI width is estimated to be 250 nm and the depth is about 2 μm suggesting well suppressed color crosstalk as shown in Fig. 6c. Still color crosstalk to adjacent pixel seems to be larger than the reference samples or penetrated DTIs judging from the simulation of Fig. 4. Although it is possible to compensate the color crosstalk to reduce color noise with signal processing, it is better to reduce the color crosstalk as small as possible to improve the visible image qualities. We would like to emphasize that it is highly required to improve pixel isolation capability to confine the light more effectively within the pixel cubic. It is also important that this structure does not degrade dark current and noise characteristics due to the uneven surface. Because geometric surface area of c-Si (111) IPA has the facet angle of 54.7° to (100), it makes the surface area increase as factor of 1.73 (=1/cos(54.7°)) compared to flat surface. However, each Si atom of c-Si (111) surface has single-dangling-bond whereas that of (100) has double-dangling-bonds, so that both the factors cancel with each other and the total number of the dangling-bond is reduced to 86.5% compared of the flat surface case. This suggests it is expected to have small degradation or might be a little bit better of dark current characteristics, if it is processed with the proper and careful surface passivation to minimize surface recombination.

## Conclusion

A novel BI-CIS with IPA on c-Si surface for light trapping pixel technology is proposed and the prototyping results are demonstrated. Both spectroscopic measurements and demo images show considerable NIR sensitivity enhancement with small spatial resolution degradation. BI-CIS with 400 nm pitch IPA surface and DTI shows 80% improvement in sensitivity, which corresponds to QE of more than 30% at 850 nm for a 3 μm thick c-Si photodetector. Furthermore, it is worth noting that there is still a lot of room for improvement toward the fundamental limit of 4*n* ^2^. Additionally, it is important to control surface passivation to minimize the degradation of thermal noise and also further improve pixel isolation to reduce lateral color crosstalk as small as possible.

## Methods

### Working principle of light trapping pixel

The brief description of the working principle of this light trapping pixel is explained in Fig. 1b. Because of the high refractive index of c-Si and low refractive index of surround medium such as SiO_2_, it is possible to effectively confine the light within the pixel cube. Firstly, incident light is refracted with the c-Si surface (111) which has facet angle of 54.7° by following Snell’s law. Then the refracted light is reflected at the side-wall and bottom of c-Si. Because of large gap of refractive index of c-Si and SiO_2_, it is easy to fulfill the total reflection condition, and results in large optical-path elongation compared to the case of flat surface.

### FDTD simulations

FDTD simulations were performed using the commercial FDTD software, Lumerical Solutions 8.5.1 with a periodic boundary condition in the XY plane and a PML condition in Z-direction^15^. For normal incidence light simulations, the structure was excited with a broadband plane-wave source which is launched 2 μm above the Si surface and steady-state electromagnetic fields were recorded to calculate amount of the absorption of c-Si within 3 μm from the surface. The broadband incidence wavelength is set to λ = 700–1000 nm in step of 10 nm for Fig. 2 and 400–800 nm in step of 20 nm for Figs 4 and 8 where the spatial mesh size were set to 2 nm in XYZ dimension for Fig. 2 and 4 nm for Figs 4 and 8. Here, 2D IPA structure has edge angle of 54.7° corresponding to the c-Si (111) surface to the (100) and are densely packed without gap.

### Fabrication of IPAs on c-Si surface of BI-CIS

Here we describe how to fabricate the IPA surface onto the Si surface of BI-CIS. We used a 1/2.3 inch, 20 M pixel BI-CIS with 1.20 μm square pixels (http://www.sony-semicon.co.jp/products_ja/new_pro/march_2014/imx147lqt_j.html) as base type. For this prototyping, we fabricated IPA composed of Si (111) using lithography and anisotropic wet etching process. At first, the PR coating and mask patterning were performed. Next 5-M potassium hydroxide (KOH) with 1-M IPA solution was used as an isotropic etchant to create c-Si (111) inverted pyramid under a carefully controlled temperature condition as KOH etching rate is sensitive to the temperature. Figure 1c,d show a cross-sectional SEM image and top-view of the inverted pyramid array with a periodicity of 400 nm and 0 nm spacing after the wet etching. The surface is passivated with a thin p + layer to minimize surface recombination and noise generation. This thin layer is also serving as anti-reflection coating. After the fabrication of the IPA structure, passivation is deposited and planarized over the pyramid array. Other processes are the same as the reference BI-CMOS process.

### Spectroscopic measurements

Spectral sensitivity measurements were performed with a calibrated halogen lamp with grating. The light power was set to be 5 μW/cm^2^ and finely collimated light was radiated onto the BI-CIS sample without optics. Although commercially available BI-CMOS image sensor chips often have some offset between the on-chip micro lenses and photo-detectors to adjust the chief-lay angle (CRA) of the camera module optics, our samples have no offset between on-chip micro lenses and photo-detectors. The image data was taken from 700 nm to 1000 nm in step of 10 nm resolution and the data was averaged for each color pixels separately by using center 1/3 part of the imager. The sensor exposure time and gain setting were 16.7 mS and 0 dB, respectively. The output data has 12 bit digit including black level offset of 200. We applied the factor of 130 μV/LSB to convert the digit to voltage to the data after subtracting the black level. The results data have spectral bandwidth of 10 nm and the absolute power error is calibrated to be less than 0.5%.
